# Crystal structure of di­aqua­bis­(*N*,*N*-di­ethyl­nicotinamide-κ*N* ^1^)bis­(2,4,6-tri­methyl­benzoato-κ*O* ^1^)cobalt(II)

**DOI:** 10.1107/S2056989016004059

**Published:** 2016-03-15

**Authors:** Gülçin Şefiye Aşkın, Hacali Necefoğlu, Safiye Özkaya, Raziye Çatak Çelik, Tuncer Hökelek

**Affiliations:** a Department of Physics, Hacettepe University, 06800 Beytepe, Ankara, Turkey; b Department of Chemistry, Kafkas University, 36100 Kars, Turkey, International Scientific Research Centre, Baku State University, 1148 Baku, Azerbaijan; c Department of Chemistry, Kafkas University, 36100 Kars, Turkey; d Scientific and Technological Application and Research Center, Aksaray University, 68100, Aksaray, Turkey

**Keywords:** crystal structure, cobalt(II), transition metal complexes of benzoic acid and nicotinamide derivatives

## Abstract

The centrosymmetric mol­ecule in the monomeric title cobalt complex, [Co(C_10_H_11_O_2_)_2_(C_10_H_14_N_2_O)_2_(H_2_O)_2_], contains two water mol­ecules, two 2,4,6-tri­methyl­benzoate (TMB) ligands and two di­ethyl­nicotinamide (DENA) ligands. All ligands coordinate to the Co^II^ atom in a monodentate fashion. The four O atoms around the Co^II^ atom form a slightly distorted square-planar arrangement, with the distorted octa­hedral coordination sphere completed by two pyridine N atoms of the DENA ligands. The dihedral angle between the planar carboxyl­ate group and the adjacent benzene ring is 84.2 (4)°, while the benzene and pyridine rings are oriented at a dihedral angle of 38.87 (10)°. The water mol­ecules exhibit both intra­molecular (to the non-coordinating carboxyl­ate O atom) and inter­molecular (to the amide carbonyl O atom) O—H⋯O hydrogen bonds. The latter lead to the formation of layers parallel to (100), enclosing *R*_4_^4^(32) ring motifs. These layers are further linked *via* weak C—H⋯O hydrogen bonds, resulting in a three-dimensional network. One of the two ethyl groups of the DENA ligand is disordered over two sets of sites with an occupancy ratio of 0.490 (13):0.510 (13).

## Abstract 2

The Co^II^ atom in the crystal structure of di­aqua­bis­(*N*,*N*-di­ethyl­nicotinamide)­bis­(2,4,6-tri­methyl­benzoato)cobalt(II) is located on an inversion centre and exhibits a slightly distorted octa­hedral N_2_O_4_ coordination set. Hydrogen bonds of the type O—H⋯O and C—H⋯O lead to the formation of a three-dimensional network.

## Chemical context   

*N*,*N*-Di­ethyl­nicotinamide (DENA), a nicotinic acid derivative, is an important respiratory stimulant (Bigoli *et al.*, 1972 ▸). The crystal structure of the complex [Co(CH_3_CO_2_)_2_(DENA)_2_(H_2_O)_2_] [(II); Mikelashvili, 1982 ▸] is isostructural with the analogous Ni, Mn, Zn and Cd complexes (Sergienko *et al.*, 1980 ▸). The structures of some complexes obtained from the reactions of transition metal(II) ions with DENA as ligand, *e.g.* [Cu_2_(DENA)_2_(C_6_H_5_COO)_4_] [(III); Hökelek *et al.*, 1995 ▸], [Zn_2_(C_7_H_5_O_3_)_4_(DENA)_2_]·2H_2_O [(IV); Hökelek & Necefoğlu, 1996 ▸], [Mn(DENA)_2_(NCS)_2_] [(V); Bigoli *et al.*, 1973*a* ▸], [Zn(DENA)_2_(NCS)_2_(H_2_O)_2_] [(VI); Bigoli *et al.*, 1973*b* ▸] and [Cd(DENA)(SCN)_2_] [(VII); Bigoli *et al.*, 1972 ▸], have been determined previously. In complex (V), DENA is a bidentate ligand, while in complexes (III), (IV), (VI) and (VII), DENA is a monodentate ligand. In complex (III), the benzoate ion acts as a bidentate ligand, whereas in complex (IV), two of the benzoate ions act as monodentate ligands, while the other two are bidentate, bridging the two Zn^II^ atoms.

The structure–function–coordination relationships of aryl­carboxyl­ate ions in Co^II^ complexes of benzoic acid derivatives may change depending on the nature and position of the substituted groups on the benzene ring, the nature of the additional ligand mol­ecule or solvent, and the pH conditions and temperature of synthesis (Shnulin *et al.*, 1981 ▸; Nadzhafov *et al.*, 1981 ▸; Antsyshkina *et al.*, 1980 ▸; Adiwidjaja *et al.*, 1978 ▸). When pyridine or its derivatives are used instead of water mol­ecules, the resulting structure is completely different (Catterick *et al.*, 1974 ▸). In this context, we synthesized a Co^II^-containing compound with 2,4,6-tri­methyl­benzoate (TMB) and DENA ligands, namely di­aqua­bis­(*N*,*N*-di­ethyl­nico­tin­amide-*κN* ^1^)bis­(2,4,6-tri­methyl­benzoato-*κO* ^1^)cobalt(II), [Co(DENA)_2_(TMB)_2_(H_2_O)_2_], and report herein its crystal structure.
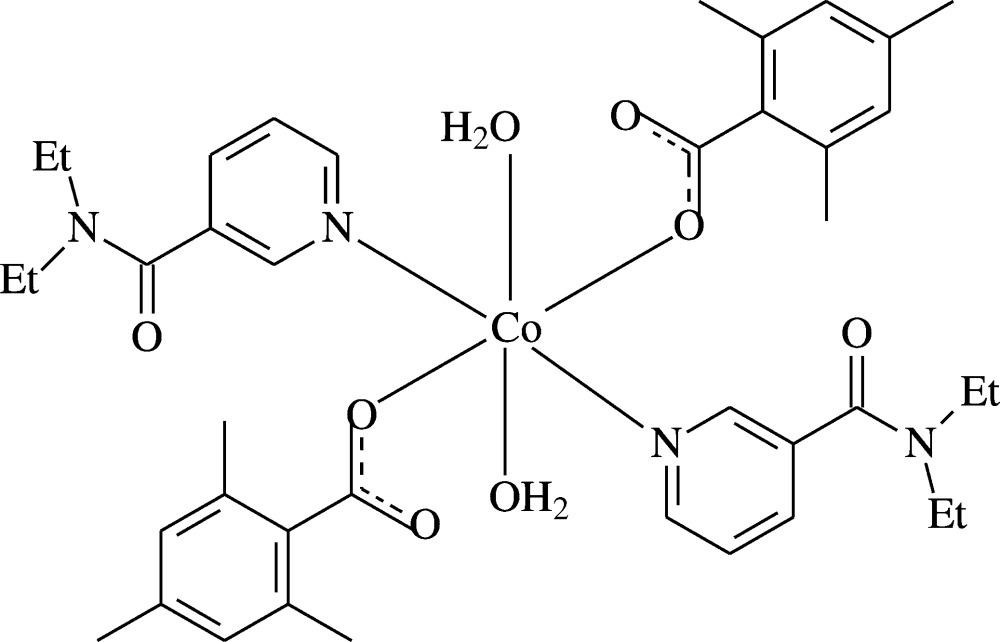


## Structural commentary   

**Figure 1:**
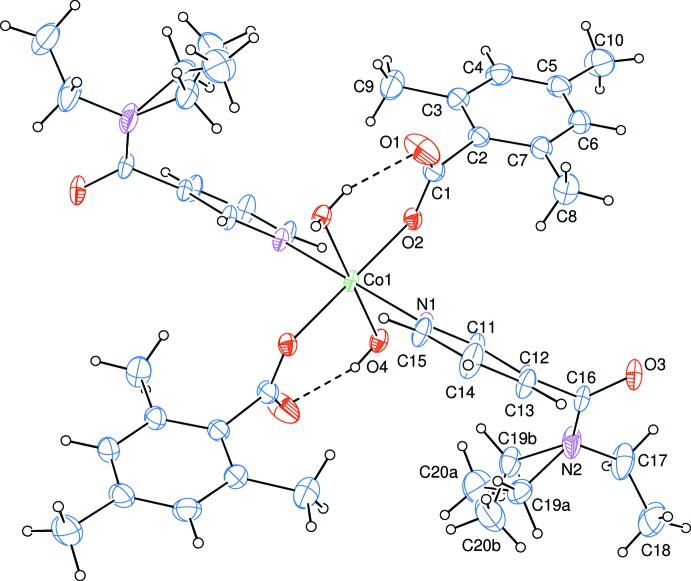
The mol­ecular structure of the title complex with the atom-numbering scheme for the asymmetric unit. Unlabelled atoms are generated by symmetry code (1 − *x*, 1 − *y*, −*z*). Displacement ellipsoids are drawn at the 40% probability level. Intra­molecular O—H⋯O hydrogen bonds are shown as dashed lines.

The asymmetric unit of the mononuclear title complex contains one Co^II^ atom located on an inversion centre, one TMB ligand, one DENA ligand and one water mol­ecule, with all ligands coordinating to the metal ion in a monodentate fashion (Fig. 1 ▸).

The two carboxyl­ate O atoms (O2 and O2^i^) of the two symmetry-related TMB anions and the two symmetry-related water O atoms (O4 and O4^i^) form a slightly distorted square-planar arrangement around the Co1 atom. The slightly distorted octa­hedral coordination sphere is completed by the two pyridine N atoms (N1 and N1^i^) of the two symmetry-related DENA ligands in axial positions [symmetry code: (i) 1 − *x*, 1 − *y*, −*z*] (Fig. 1 ▸). The Co—O bond lengths for water oxygens atoms are by *ca* 0.1 Å longer than those involving the benzoate oxygen atoms. The Co—N bond length is the longest in the CoO_4_N_2_ octa­hedron (Table 1 ▸). The deviation of the O—Co—O and O—Co—N bond angles from ideal values is minute [range 87.66 (7) to 92.34 (7)° for *cis* angles; all *trans* angles are 180° due to symmetry]. The near equalities of the C1—O1 [1.245 (4) Å] and C1—O2 [1.254 (4) Å] bonds in the carboxyl­ate group indicate delocalized bonding arrangements, rather than localized single and double bonds. The dihedral angle between the planar carboxyl­ate group (O1/O2/C1) and the adjacent benzene ring *A* (C2–C7) is 84.2 (4)°, while the benzene (*A*) and pyridine rings (*B*) (N1/C11–C15) are inclined by a dihedral angle of 38.87 (10)°.

## Supra­molecular features   

**Figure 2:**
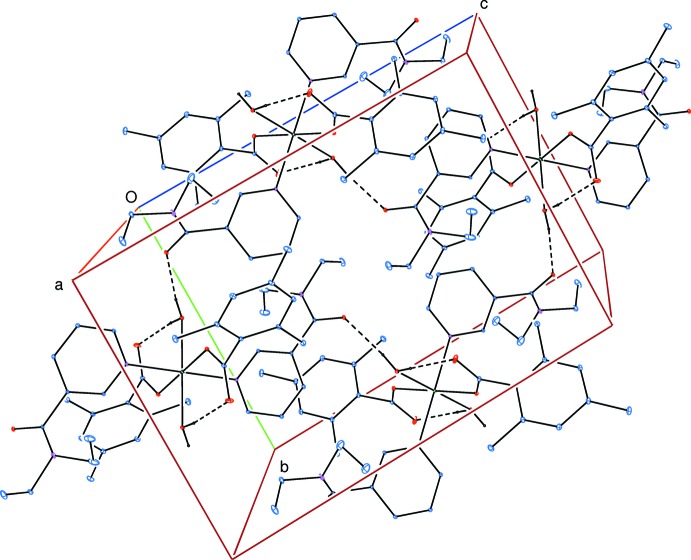
Part of the crystal structure viewed approximately down [100]. Intra- and inter­molecular O—H⋯O hydrogen bonds, shown as dashed lines, enclose 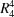
(32) ring motifs. Only one part of the disordered group and only H atoms involved in hydrogen bonding have been included for clarity.

**Figure 3:**
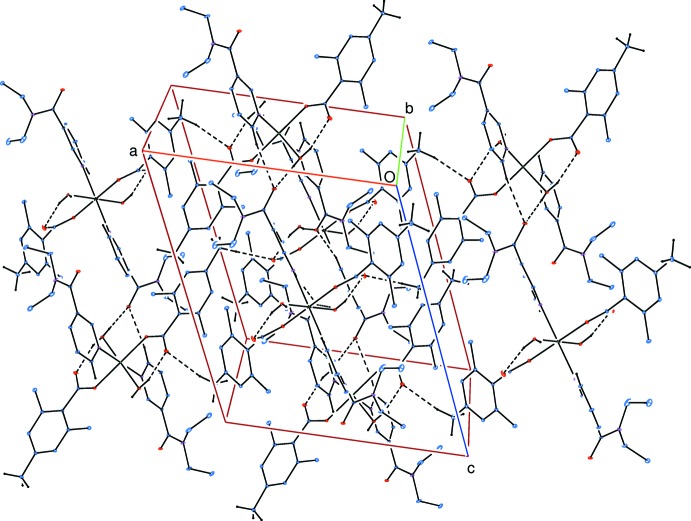
A partial view of the crystal packing of the title compound. The O—H_w_⋯O_c_, O—H_w_⋯O_DENA_, C—H_TMB_⋯O_c_ and C—H_DENA_⋯O_DENA_ (w = water, c = carboxyl­ate, DENA = *N*,*N*-di­ethyl­nicotinamide and TMB = 2,4,6-tri­methyl­benzoate) hydrogen bonds, enclosing 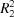
(7) and 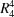
(32) ring motifs, are shown as dashed lines (see Table 2 ▸). Only one part of the disordered group and only H atoms involved in hydrogen bonding have been included for clarity.

Intra­molecular O—H_w_⋯O_c_ (w = water, c = non-coordinating carboxyl­ate O atom) hydrogen bonds (Table 2 ▸) link the water ligands to the TMB anions (Fig. 1 ▸). The other water H atom is involved in inter­molecular O—H_w_⋯O_DENA_ (O_DENA_ = carbonyl O atom of *N*,*N*-di­ethyl­nicotinamide) hydrogen bonds (Table 2 ▸), leading to the formation of layers parallel to (100) enclosing 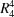
(32) ring motifs (Fig. 2 ▸). The layers are further linked into a three-dimensional network structure *via* weak C—H_TMB_⋯O_c_ (TMB = 2,4,6-tri­methyl­benzoate) and C—H_DENA_ ⋯ O_DENA_ hydrogen bonds (Table 2 ▸), enclosing 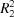
(7) ring motifs (Fig. 3 ▸).

## Synthesis and crystallization   

The title compound was prepared by the reaction of CoSO_4_·7H_2_O (1.41 g, 5 mmol) in H_2_O (100 ml) and *N,N*-di­ethyl­nicotinamide (1.78 g, 10 mmol) in H_2_O (10 ml) with sodium 2,4,6-tri­methyl­benzoate (1.86 g, 10 mmol) in H_2_O (150 ml). The mixture was filtered and set aside to crystallize at ambient temperature for three weeks, giving pink single crystals.

## Refinement   

Experimental details including crystal data, data collection and refinement are summarized in Table 3 ▸. Atoms H1*W* and H2*W* (of the water molecule) were located in a difference Fourier map. Their coordinates were refined freely, with *U*_iso_(H) = 1.5*U*_eq_(O). C-bound H atoms were positioned geometrically, with C—H = 0.93, 0.96 and 0.97 Å for aromatic, methyl and methyl­ene H atoms, respectively, and constrained to ride on their parent atoms, with *U*_iso_(H) = *k* × *U*_eq_(C), where *k* = 1.5 for methyl H atoms and *k* = 1.2 for other H atoms. The disordered ethyl group (C19, C20) was refined over two sets of sites with distance restraints and SIMU and DELU restraints (Sheldrick, 2008 ▸). The refined occupancy ratio of the two orientations is 0.490 (13):0.510 (13).

## Supplementary Material

Crystal structure: contains datablock(s) I, global. DOI: 10.1107/S2056989016004059/wm5273sup1.cif

Structure factors: contains datablock(s) I. DOI: 10.1107/S2056989016004059/wm5273Isup2.hkl

CCDC reference: 1462884

Additional supporting information:  crystallographic information; 3D view; checkCIF report

## Figures and Tables

**Table 1:** Selected bond lengths (Å)

Co1—O2	2.0336 (18)	Co1—N1	2.1913 (19)
Co1—O4	2.1561 (18)		

**Table 2:** Hydrogen-bond geometry (Å, °)

*D*—H⋯*A*	*D*—H	H⋯*A*	*D*⋯*A*	*D*—H⋯*A*
O4—H1*W*⋯O1^i^	0.80 (6)	1.87 (6)	2.634 (3)	160 (7)
O4—H2*W*⋯O3^ii^	0.76 (7)	2.10 (7)	2.850 (3)	170 (7)
C10—H10*A*⋯O1^iii^	0.96	2.43	3.365 (6)	165
C15—H15⋯O3^iv^	0.93	2.50	3.420 (4)	172

Symmetry codes: (i) 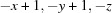; (ii) 
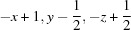
; (iii) 
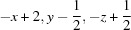
; (iv) 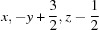.

**Table 3:** Experimental details

Crystal data	
Chemical formula	[Co(C_10_H_11_O_2_)_2_(C_10_H_14_N_2_O)_2_(H_2_O)_2_]
*M*_r_	777.80
Crystal system, space group	Monoclinic, *P*2_1_/*c*
Temperature (K)	100
*a*, *b*, *c* (Å)	12.9646 (4), 10.8636 (3), 15.6297 (5)
β (°)	111.596 (3)
*V* (Å^3^)	2046.79 (12)
*Z*	2
Radiation type	Mo *K*α
μ (mm^−1^)	0.47
Crystal size (mm)	0.45 × 0.40 × 0.33

Data collection	
Diffractometer	Bruker SMART BREEZE CCD
Absorption correction	Multi-scan (*SADABS*; Bruker, 2012 ▸)
*T*_min_, *T*_max_	0.754, 0.861
No. of measured, independent and observed [*I* > 2σ(*I*)] reflections	42492, 5124, 3701
*R*_int_	0.041
(sin θ/λ)_max_ (Å^−1^)	0.670

Refinement	
*R*[*F* ^2^ > 2σ(*F* ^2^)], *wR*(*F* ^2^), *S*	0.063, 0.155, 1.07
No. of reflections	5124
No. of parameters	270
No. of restraints	42
H-atom treatment	H atoms treated by a mixture of independent and constrained refinement
Δρ_max_, Δρ_min_ (e Å^−3^)	0.63, −0.39

Computer programs: *APEX2* and *SAINT* (Bruker, 2012 ▸), *SHELXS97* and *SHELXL97* (Sheldrick, 2008 ▸), *ORTEP-3 for Windows* (Farrugia, 2012 ▸), *WinGX* (Farrugia, 2012 ▸) and *PLATON* (Spek, 2009 ▸).

## supplementary crystallographic information

### Crystal data

**Table:**

[Co(C_10_H_11_O_2_)_2_(C_10_H_14_N_2_O)_2_(H_2_O)_2_]	*F*(000) = 826
*M**_r_* = 777.80	*D*_x_ = 1.262 Mg m^−^^3^
Monoclinic, *P*2_1_/*c*	Mo *K*α radiation, λ = 0.71073 Å
Hall symbol: -P 2ybc	Cell parameters from 9990 reflections
*a* = 12.9646 (4) Å	θ = 2.3–28.4°
*b* = 10.8636 (3) Å	µ = 0.47 mm^−^^1^
*c* = 15.6297 (5) Å	*T* = 100 K
β = 111.596 (3)°	Block, translucent light pink
*V* = 2046.79 (12) Å^3^	0.45 × 0.40 × 0.33 mm
*Z* = 2	

### Data collection

**Table:**

Bruker SMART BREEZE CCD diffractometer	5124 independent reflections
Radiation source: fine-focus sealed tube	3701 reflections with *I* > 2σ(*I*)
Graphite monochromator	*R*_int_ = 0.041
φ and ω scans	θ_max_ = 28.5°, θ_min_ = 1.7°
Absorption correction: multi-scan (*SADABS*; Bruker, 2012)	*h* = −17→16
*T*_min_ = 0.754, *T*_max_ = 0.861	*k* = −14→14
42492 measured reflections	*l* = −20→20

### Refinement

**Table:**

Refinement on *F*^2^	Primary atom site location: structure-invariant direct methods
Least-squares matrix: full	Secondary atom site location: difference Fourier map
*R*[*F*^2^ > 2σ(*F*^2^)] = 0.063	Hydrogen site location: inferred from neighbouring sites
*wR*(*F*^2^) = 0.155	H atoms treated by a mixture of independent and constrained refinement
*S* = 1.07	*w* = 1/[σ^2^(*F*_o_^2^) + (0.0662*P*)^2^ + 1.2728*P*] where *P* = (*F*_o_^2^ + 2*F*_c_^2^)/3
5124 reflections	(Δ/σ)_max_ < 0.001
270 parameters	Δρ_max_ = 0.63 e Å^−^^3^
42 restraints	Δρ_min_ = −0.39 e Å^−^^3^

### Special details

**Table:**

Geometry. All esds (except the esd in the dihedral angle between two l.s. planes) are estimated using the full covariance matrix. The cell esds are taken into account individually in the estimation of esds in distances, angles and torsion angles; correlations between esds in cell parameters are only used when they are defined by crystal symmetry. An approximate (isotropic) treatment of cell esds is used for estimating esds involving l.s. planes.
Refinement. Refinement of F^2^ against ALL reflections. The weighted R-factor wR and goodness of fit S are based on F^2^, conventional R-factors R are based on F, with F set to zero for negative F^2^. The threshold expression of F^2^ > 2sigma(F^2^) is used only for calculating R-factors(gt) etc. and is not relevant to the choice of reflections for refinement. R-factors based on F^2^ are statistically about twice as large as those based on F, and R- factors based on ALL data will be even larger.

### Fractional atomic coordinates and isotropic or equivalent isotropic displacement parameters (Å^2^)

**Table:**

	*x*	*y*	*z*	*U*_iso_*/*U*_eq_	Occ. (<1)
Co1	0.5000	0.5000	0.0000	0.03723 (16)	
O1	0.7500 (2)	0.6149 (3)	0.1133 (2)	0.1140 (12)	
O2	0.64234 (15)	0.45260 (17)	0.10503 (11)	0.0476 (4)	
O3	0.47496 (19)	0.62630 (18)	0.39053 (11)	0.0613 (5)	
O4	0.41728 (17)	0.35512 (17)	0.04417 (13)	0.0495 (5)	
H1W	0.358 (6)	0.358 (7)	0.004 (5)	0.201*	
H2W	0.440 (6)	0.290 (6)	0.057 (5)	0.201*	
N1	0.45672 (18)	0.62382 (18)	0.09231 (13)	0.0434 (5)	
N2	0.3331 (3)	0.5092 (3)	0.30486 (19)	0.0929 (12)	
C1	0.7318 (3)	0.5109 (3)	0.1382 (2)	0.0589 (8)	
C2	0.8208 (2)	0.4486 (3)	0.21743 (19)	0.0546 (7)	
C3	0.8883 (3)	0.3599 (3)	0.2011 (2)	0.0684 (9)	
C4	0.9635 (3)	0.2965 (4)	0.2757 (3)	0.0784 (10)	
H4	1.0091	0.2369	0.2654	0.094*	
C5	0.9713 (3)	0.3208 (4)	0.3649 (2)	0.0761 (10)	
C6	0.9055 (3)	0.4105 (4)	0.3785 (2)	0.0713 (9)	
H6	0.9109	0.4279	0.4383	0.086*	
C7	0.8310 (3)	0.4766 (3)	0.3068 (2)	0.0621 (8)	
C8	0.7606 (4)	0.5758 (4)	0.3247 (3)	0.0895 (12)	
H8A	0.7634	0.5690	0.3867	0.134*	
H8B	0.6853	0.5668	0.2827	0.134*	
H8C	0.7882	0.6550	0.3161	0.134*	
C9	0.8814 (4)	0.3334 (5)	0.1040 (3)	0.1066 (15)	
H9A	0.9321	0.2684	0.1052	0.160*	
H9B	0.9005	0.4062	0.0783	0.160*	
H9C	0.8072	0.3087	0.0670	0.160*	
C10	1.0488 (4)	0.2448 (5)	0.4441 (3)	0.1131 (16)	
H10A	1.1133	0.2225	0.4313	0.170*	
H10B	1.0112	0.1717	0.4513	0.170*	
H10C	1.0709	0.2923	0.4998	0.170*	
C11	0.4479 (2)	0.5794 (2)	0.16908 (15)	0.0457 (6)	
H11	0.4627	0.4964	0.1823	0.055*	
C12	0.4181 (2)	0.6499 (2)	0.22983 (15)	0.0425 (6)	
C13	0.4001 (3)	0.7736 (3)	0.21270 (19)	0.0602 (8)	
H13	0.3822	0.8244	0.2531	0.072*	
C14	0.4095 (3)	0.8204 (3)	0.1337 (2)	0.0735 (10)	
H14	0.3980	0.9038	0.1201	0.088*	
C15	0.4357 (3)	0.7430 (3)	0.07530 (18)	0.0574 (7)	
H15	0.4390	0.7755	0.0213	0.069*	
C16	0.4100 (3)	0.5942 (2)	0.31519 (16)	0.0501 (7)	
C17	0.3272 (4)	0.4478 (4)	0.3879 (3)	0.1070 (16)	
H17A	0.3127	0.3608	0.3753	0.128*	
H17B	0.3983	0.4558	0.4381	0.128*	
C18	0.2407 (5)	0.5002 (6)	0.4159 (4)	0.154 (3)	
H18A	0.2431	0.4618	0.4719	0.231*	
H18B	0.1695	0.4862	0.3685	0.231*	
H18C	0.2528	0.5871	0.4258	0.231*	
C19A	0.2227 (8)	0.5259 (10)	0.2181 (7)	0.080 (3)	0.490 (13)
H19A	0.1601	0.5419	0.2364	0.096*	0.490 (13)
H19B	0.2294	0.5928	0.1794	0.096*	0.490 (13)
C19B	0.2696 (8)	0.4402 (8)	0.2180 (5)	0.077 (3)	0.510 (13)
H19C	0.2477	0.3595	0.2318	0.093*	0.510 (13)
H19D	0.3135	0.4307	0.1798	0.093*	0.510 (13)
C20A	0.2102 (12)	0.4038 (11)	0.1694 (9)	0.128 (5)	0.490 (13)
H20A	0.1443	0.4049	0.1148	0.192*	0.490 (13)
H20B	0.2047	0.3392	0.2094	0.192*	0.490 (13)
H20C	0.2736	0.3896	0.1529	0.192*	0.490 (13)
C20B	0.1696 (11)	0.5203 (13)	0.1714 (9)	0.134 (5)	0.510 (13)
H20D	0.1236	0.4828	0.1146	0.201*	0.510 (13)
H20E	0.1933	0.5999	0.1592	0.201*	0.510 (13)
H20F	0.1281	0.5292	0.2109	0.201*	0.510 (13)

### Atomic displacement parameters (Å^2^)

**Table:**

	*U*^11^	*U*^22^	*U*^33^	*U*^12^	*U*^13^	*U*^23^
Co1	0.0546 (3)	0.0354 (2)	0.0260 (2)	−0.0004 (2)	0.0197 (2)	0.00073 (17)
O1	0.0881 (19)	0.100 (2)	0.115 (2)	−0.0333 (16)	−0.0081 (16)	0.0591 (18)
O2	0.0552 (11)	0.0477 (10)	0.0375 (9)	−0.0020 (9)	0.0143 (8)	0.0047 (8)
O3	0.1013 (16)	0.0519 (11)	0.0305 (9)	−0.0127 (11)	0.0240 (10)	−0.0057 (8)
O4	0.0709 (13)	0.0412 (10)	0.0428 (10)	−0.0004 (9)	0.0287 (9)	0.0037 (8)
N1	0.0644 (14)	0.0388 (11)	0.0315 (10)	0.0001 (10)	0.0231 (10)	0.0017 (8)
N2	0.121 (3)	0.122 (3)	0.0444 (14)	−0.066 (2)	0.0413 (17)	−0.0121 (15)
C1	0.0588 (18)	0.0664 (19)	0.0506 (16)	−0.0066 (15)	0.0188 (14)	0.0134 (14)
C2	0.0478 (16)	0.0628 (17)	0.0507 (15)	−0.0064 (14)	0.0154 (13)	0.0107 (13)
C3	0.0615 (19)	0.085 (2)	0.0600 (18)	−0.0051 (18)	0.0241 (16)	0.0060 (17)
C4	0.059 (2)	0.093 (3)	0.086 (2)	0.0135 (19)	0.0313 (19)	0.016 (2)
C5	0.0547 (19)	0.100 (3)	0.067 (2)	0.0009 (18)	0.0144 (16)	0.0269 (19)
C6	0.063 (2)	0.095 (3)	0.0503 (17)	−0.0042 (19)	0.0149 (15)	0.0123 (17)
C7	0.0588 (18)	0.072 (2)	0.0528 (17)	−0.0072 (15)	0.0173 (14)	0.0084 (14)
C8	0.108 (3)	0.088 (3)	0.074 (2)	0.013 (2)	0.036 (2)	0.002 (2)
C9	0.112 (3)	0.142 (4)	0.076 (3)	0.014 (3)	0.046 (3)	−0.005 (3)
C10	0.087 (3)	0.147 (4)	0.095 (3)	0.028 (3)	0.021 (2)	0.054 (3)
C11	0.0712 (18)	0.0384 (13)	0.0309 (11)	−0.0009 (12)	0.0229 (12)	0.0006 (9)
C12	0.0573 (15)	0.0439 (13)	0.0293 (11)	−0.0054 (11)	0.0193 (11)	−0.0032 (9)
C13	0.095 (2)	0.0503 (16)	0.0467 (14)	0.0119 (15)	0.0400 (16)	−0.0028 (12)
C14	0.136 (3)	0.0410 (15)	0.0594 (17)	0.0231 (17)	0.054 (2)	0.0102 (13)
C15	0.095 (2)	0.0469 (15)	0.0424 (13)	0.0085 (15)	0.0403 (15)	0.0102 (12)
C16	0.0799 (19)	0.0448 (14)	0.0334 (12)	−0.0066 (14)	0.0300 (13)	−0.0068 (10)
C17	0.167 (5)	0.104 (3)	0.069 (2)	−0.057 (3)	0.065 (3)	−0.003 (2)
C18	0.151 (5)	0.246 (8)	0.095 (4)	−0.066 (5)	0.080 (4)	−0.016 (4)
C19A	0.077 (7)	0.100 (7)	0.077 (6)	−0.026 (6)	0.043 (5)	−0.015 (5)
C19B	0.110 (6)	0.068 (5)	0.064 (5)	−0.040 (5)	0.043 (4)	−0.023 (4)
C20A	0.129 (10)	0.123 (9)	0.129 (9)	−0.050 (8)	0.045 (8)	−0.064 (8)
C20B	0.110 (9)	0.165 (13)	0.097 (9)	−0.019 (7)	0.004 (7)	−0.011 (8)

### Geometric parameters (Å, º)

**Table:**

Co1—O2	2.0336 (18)	C9—H9C	0.9600
Co1—O2^i^	2.0336 (18)	C10—H10A	0.9600
Co1—O4	2.1561 (18)	C10—H10B	0.9600
Co1—O4^i^	2.1561 (19)	C10—H10C	0.9600
Co1—N1	2.1913 (19)	C11—H11	0.9300
Co1—N1^i^	2.1913 (19)	C12—C11	1.381 (3)
O2—C1	1.254 (4)	C12—C16	1.503 (3)
O4—H1W	0.80 (7)	C13—C12	1.374 (4)
O4—H2W	0.76 (7)	C13—C14	1.382 (4)
N1—C11	1.336 (3)	C13—H13	0.9300
N1—C15	1.330 (3)	C14—C15	1.372 (4)
N2—C17	1.486 (4)	C14—H14	0.9300
N2—C19A	1.579 (11)	C15—H15	0.9300
N2—C19B	1.503 (8)	C16—N2	1.325 (4)
C1—O1	1.245 (4)	C17—H17A	0.9700
C1—C2	1.508 (4)	C17—H17B	0.9700
C2—C7	1.387 (4)	C18—C17	1.462 (7)
C3—C2	1.387 (5)	C18—H18A	0.9600
C3—C4	1.396 (5)	C18—H18B	0.9600
C3—C9	1.514 (5)	C18—H18C	0.9600
C4—H4	0.9300	C19A—C20A	1.508 (13)
C5—C4	1.385 (5)	C19A—H19A	0.9700
C5—C10	1.519 (5)	C19A—H19B	0.9700
C6—C5	1.363 (5)	C19B—C20B	1.508 (14)
C6—H6	0.9300	C19B—H19C	0.9700
C7—C6	1.381 (4)	C19B—H19D	0.9700
C7—C8	1.504 (5)	C20A—H20A	0.9600
C8—H8A	0.9600	C20A—H20B	0.9600
C8—H8B	0.9600	C20A—H20C	0.9600
C8—H8C	0.9600	C20B—H20D	0.9600
C9—H9A	0.9600	C20B—H20E	0.9600
C9—H9B	0.9600	C20B—H20F	0.9600
			
O2^i^—Co1—O2	180.00 (13)	C5—C10—H10A	109.5
O2—Co1—O4	88.12 (7)	C5—C10—H10B	109.5
O2^i^—Co1—O4	91.88 (7)	C5—C10—H10C	109.5
O2—Co1—O4^i^	91.88 (7)	H10A—C10—H10B	109.5
O2^i^—Co1—O4^i^	88.12 (7)	H10A—C10—H10C	109.5
O2—Co1—N1	90.01 (8)	H10B—C10—H10C	109.5
O2^i^—Co1—N1	89.99 (8)	N1—C11—C12	123.7 (2)
O2—Co1—N1^i^	89.99 (8)	N1—C11—H11	118.1
O2^i^—Co1—N1^i^	90.01 (8)	C12—C11—H11	118.1
O4—Co1—O4^i^	180.00 (9)	C11—C12—C16	121.1 (2)
O4—Co1—N1	87.66 (7)	C13—C12—C11	118.6 (2)
O4^i^—Co1—N1	92.34 (7)	C13—C12—C16	120.3 (2)
O4—Co1—N1^i^	92.34 (7)	C12—C13—C14	118.0 (2)
O4^i^—Co1—N1^i^	87.66 (7)	C12—C13—H13	121.0
N1—Co1—N1^i^	180.00 (7)	C13—C14—H14	120.2
C1—O2—Co1	129.21 (18)	C14—C13—H13	121.0
Co1—O4—H1W	99 (5)	C15—C14—C13	119.6 (3)
Co1—O4—H2W	125 (5)	C15—C14—H14	120.2
H1W—O4—H2W	115 (6)	N1—C15—C14	123.1 (2)
C11—N1—Co1	119.79 (16)	N1—C15—H15	118.5
C15—N1—Co1	123.32 (16)	C14—C15—H15	118.5
C15—N1—C11	116.9 (2)	O3—C16—N2	122.6 (2)
C16—N2—C17	119.0 (3)	O3—C16—C12	119.6 (2)
C16—N2—C19A	115.4 (4)	N2—C16—C12	117.9 (2)
C16—N2—C19B	126.5 (3)	N2—C17—H17A	109.1
C17—N2—C19A	118.7 (4)	N2—C17—H17B	109.1
C17—N2—C19B	112.2 (4)	C18—C17—N2	112.3 (5)
O1—C1—O2	125.6 (3)	C18—C17—H17A	109.1
O1—C1—C2	119.2 (3)	C18—C17—H17B	109.1
O2—C1—C2	115.1 (2)	H17A—C17—H17B	107.9
C3—C2—C1	120.3 (3)	C17—C18—H18A	109.5
C3—C2—C7	120.4 (3)	C17—C18—H18B	109.5
C7—C2—C1	119.3 (3)	C17—C18—H18C	109.5
C2—C3—C4	118.8 (3)	H18A—C18—H18B	109.5
C2—C3—C9	120.6 (3)	H18A—C18—H18C	109.5
C4—C3—C9	120.6 (4)	H18B—C18—H18C	109.5
C3—C4—H4	119.4	N2—C19A—H19A	111.1
C5—C4—C3	121.1 (3)	N2—C19A—H19B	111.1
C5—C4—H4	119.4	C20A—C19A—N2	103.1 (10)
C4—C5—C10	119.9 (4)	C20A—C19A—H19A	111.1
C6—C5—C4	118.4 (3)	C20A—C19A—H19B	111.1
C6—C5—C10	121.7 (4)	H19A—C19A—H19B	109.1
C5—C6—C7	122.4 (3)	N2—C19B—C20B	103.7 (9)
C5—C6—H6	118.8	N2—C19B—H19C	111.0
C7—C6—H6	118.8	N2—C19B—H19D	111.0
C2—C7—C8	120.4 (3)	C20B—C19B—H19C	111.0
C6—C7—C2	118.8 (3)	C20B—C19B—H19D	111.0
C6—C7—C8	120.8 (3)	H19C—C19B—H19D	109.0
C7—C8—H8A	109.5	C19A—C20A—H20A	109.5
C7—C8—H8B	109.5	C19A—C20A—H20B	109.5
C7—C8—H8C	109.5	C19A—C20A—H20C	109.5
H8A—C8—H8B	109.5	H20A—C20A—H20B	109.5
H8A—C8—H8C	109.5	H20A—C20A—H20C	109.5
H8B—C8—H8C	109.5	H20B—C20A—H20C	109.5
C3—C9—H9A	109.5	C19B—C20B—H20D	109.5
C3—C9—H9B	109.5	C19B—C20B—H20E	109.5
C3—C9—H9C	109.5	C19B—C20B—H20F	109.5
H9A—C9—H9B	109.5	H20D—C20B—H20E	109.5
H9A—C9—H9C	109.5	H20D—C20B—H20F	109.5
H9B—C9—H9C	109.5	H20E—C20B—H20F	109.5
			
O4—Co1—O2—C1	−165.8 (2)	C1—C2—C7—C8	−5.2 (5)
O4^i^—Co1—O2—C1	14.2 (2)	C3—C2—C7—C6	−3.2 (5)
N1—Co1—O2—C1	−78.1 (2)	C3—C2—C7—C8	177.8 (3)
N1^i^—Co1—O2—C1	101.9 (2)	C4—C3—C2—C1	−174.6 (3)
O2—Co1—N1—C11	−57.7 (2)	C4—C3—C2—C7	2.4 (5)
O2^i^—Co1—N1—C11	122.3 (2)	C9—C3—C2—C1	6.1 (5)
O2—Co1—N1—C15	123.9 (2)	C9—C3—C2—C7	−177.0 (3)
O2^i^—Co1—N1—C15	−56.1 (2)	C2—C3—C4—C5	0.0 (5)
O4—Co1—N1—C11	30.5 (2)	C9—C3—C4—C5	179.4 (4)
O4^i^—Co1—N1—C11	−149.5 (2)	C6—C5—C4—C3	−1.5 (6)
O4—Co1—N1—C15	−148.0 (2)	C10—C5—C4—C3	176.2 (4)
O4^i^—Co1—N1—C15	32.0 (2)	C7—C6—C5—C4	0.7 (5)
Co1—O2—C1—O1	2.1 (5)	C7—C6—C5—C10	−177.0 (4)
Co1—O2—C1—C2	179.24 (18)	C2—C7—C6—C5	1.6 (5)
Co1—N1—C11—C12	−178.2 (2)	C8—C7—C6—C5	−179.4 (3)
C15—N1—C11—C12	0.4 (4)	C13—C12—C11—N1	−2.4 (4)
Co1—N1—C15—C14	−179.5 (3)	C16—C12—C11—N1	−179.8 (3)
C11—N1—C15—C14	2.0 (5)	C11—C12—C16—O3	113.7 (3)
C16—N2—C17—C18	99.5 (5)	C11—C12—C16—N2	−64.8 (4)
C19A—N2—C17—C18	−49.9 (7)	C13—C12—C16—O3	−63.7 (4)
C19B—N2—C17—C18	−96.4 (6)	C13—C12—C16—N2	117.9 (4)
C16—N2—C19A—C20A	125.7 (6)	C14—C13—C12—C11	2.0 (5)
C17—N2—C19A—C20A	−83.8 (7)	C14—C13—C12—C16	179.5 (3)
C19B—N2—C19A—C20A	8.4 (7)	C12—C13—C14—C15	0.2 (5)
C16—N2—C19B—C20B	−89.3 (8)	C13—C14—C15—N1	−2.3 (6)
C17—N2—C19B—C20B	108.1 (8)	O3—C16—N2—C17	−1.6 (6)
C19A—N2—C19B—C20B	−0.6 (9)	O3—C16—N2—C19A	148.8 (5)
O1—C1—C2—C3	−99.3 (4)	O3—C16—N2—C19B	−163.2 (6)
O1—C1—C2—C7	83.8 (4)	C12—C16—N2—C17	176.8 (3)
O2—C1—C2—C3	83.4 (4)	C12—C16—N2—C19A	−32.8 (5)
O2—C1—C2—C7	−93.6 (4)	C12—C16—N2—C19B	15.2 (7)
C1—C2—C7—C6	173.8 (3)		

Symmetry code: (i) −*x*+1, −*y*+1, −*z*.

### Hydrogen-bond geometry (Å, º)

**Table:**

*D*—H···*A*	*D*—H	H···*A*	*D*···*A*	*D*—H···*A*
O4—H1*W*···O1^i^	0.80 (6)	1.87 (6)	2.634 (3)	160 (7)
O4—H2*W*···O3^ii^	0.76 (7)	2.10 (7)	2.850 (3)	170 (7)
C10—H10*A*···O1^iii^	0.96	2.43	3.365 (6)	165
C15—H15···O3^iv^	0.93	2.50	3.420 (4)	172

Symmetry codes: (i) −*x*+1, −*y*+1, −*z*; (ii) −*x*+1, *y*−1/2, −*z*+1/2; (iii) −*x*+2, *y*−1/2, −*z*+1/2; (iv) *x*, −*y*+3/2, *z*−1/2.

## References

[bb1] Adiwidjaja, G., Rossmanith, E. & Küppers, H. (1978). *Acta Cryst.* B**34**, 3079–3083.

[bb2] Antsyshkina, A. S., Chiragov, F. M. & Poray-Koshits, M. A. (1980). *Koord. Khim.* **15**, 1098–1103.

[bb3] Bigoli, F., Braibanti, A., Pellinghelli, M. A. & Tiripicchio, A. (1972). *Acta Cryst.* B**28**, 962–966.

[bb4] Bigoli, F., Braibanti, A., Pellinghelli, M. A. & Tiripicchio, A. (1973*a*). *Acta Cryst.* B**29**, 39–43.

[bb5] Bigoli, F., Braibanti, A., Pellinghelli, M. A. & Tiripicchio, A. (1973*b*). *Acta Cryst.* B**29**, 2344–2348.

[bb6] Bruker (2012). *APEX2*, *SAINT* and *SADABS*. Bruker AXS Inc. Madison, Wisconsin, USA.

[bb7] Catterick (neé Drew), J., Hursthouse, M. B., New, D. B. & Thornton, P. (1974). *J. Chem. Soc. Chem. Commun.* pp. 843–844.

[bb8] Farrugia, L. J. (2012). *J. Appl. Cryst.* **45**, 849–854.

[bb9] Hökelek, T. & Necefoğlu, H. (1996). *Acta Cryst.* C**52**, 1128–1131.

[bb10] Hökelek, T., Necefoğlu, H. & Balcı, M. (1995). *Acta Cryst.* C**51**, 2020–2023.

[bb11] Mikelashvili, Z. A. (1982). Dissertation, Tbilisi State University, Georgia.

[bb12] Nadzhafov, G. N., Shnulin, A. N. & Mamedov, Kh. S. (1981). *Zh. Strukt. Khim.* **22**, 124–128.

[bb13] Sergienko, V. S., Shurkina, V. N., Khodashova, T. S., Poray-Koshits, M. A. & Tsintsadze, G. V. (1980). *Koord. Khim.* **6**, 1606–1609.

[bb14] Sheldrick, G. M. (2008). *Acta Cryst.* A**64**, 112–122. 10.1107/S0108767307043930 18156677

[bb15] Shnulin, A. N., Nadzhafov, G. N., Amiraslanov, I. R., Usubaliev, B. T. & Mamedov, Kh. S. (1981). *Koord. Khim.* **7**, 1409–1416.

[bb16] Spek, A. L. (2009). *Acta Cryst.* D**65**, 148–155. 10.1107/S090744490804362X PMC2631630 19171970

